# The Interference between SARS-CoV-2 and Tyrosine Kinase Receptor Signaling in Cancer

**DOI:** 10.3390/ijms22094830

**Published:** 2021-05-02

**Authors:** Oana-Stefana Purcaru, Stefan-Alexandru Artene, Edmond Barcan, Cristian Adrian Silosi, Ilona Stanciu, Suzana Danoiu, Stefania Tudorache, Ligia Gabriela Tataranu, Anica Dricu

**Affiliations:** 1 Department of Biochemistry, Faculty of Medicine, University of Medicine and Pharmacy of Craiova, Str. Petru Rares nr. 2-4, 710204 Craiova, Romania; stoapo@yahoo.com (O.-S.P.); stefan.artene@yahoo.com (S.-A.A.); edmond.barcan@gmail.com (E.B.); anica.dricu@live.co.uk (A.D.); 2 Department of Surgery, Faculty of Medicine, University of Medicine and Pharmacy of Craiova, Str. Petru Rares nr. 2-4, 710204 Craiova, Romania; cristian_silosi@yahoo.fr; 3 “Victor Babeş” Clinical Hospital of Infectious Diseases and Pneumophtisiology, Craiova, Str. Calea Bucuresti, nr. 126, 200525 Craiova, Romania; stanciuilona@yahoo.com; 4 Department of Physiopathology, Faculty of Medicine, University of Medicine and Pharmacy of Craiova, Str. Petru Rares nr. 2-4, 710204 Craiova, Romania; suzanadanoiu@yahoo.com; 5 Department of Obstetrics and Gynecology, University of Medicine and Pharmacy Craiova, 710204 Craiova, Romania; stefania.tudorache@gmail.com; 6 Department of Neurosurgery, “Bagdasar-Arseni” Emergency Hospital, Soseaua Berceni 12, 041915 Bucharest, Romania

**Keywords:** coronavirus, pandemic, tyrosine kinase, receptor, signaling pathway, EGFR

## Abstract

Cancer and viruses have a long history that has evolved over many decades. Much information about the interplay between viruses and cell proliferation and metabolism has come from the history of clinical cases of patients infected with virus-induced cancer. In addition, information from viruses used to treat some types of cancer is valuable. Now, since the global coronavirus pandemic erupted almost a year ago, the scientific community has invested countless time and resources to slow down the infection rate and diminish the number of casualties produced by this highly infectious pathogen. A large percentage of cancer cases diagnosed are strongly related to dysregulations of the tyrosine kinase receptor (TKR) family and its downstream signaling pathways. As such, many therapeutic agents have been developed to strategically target these structures in order to hinder certain mechanisms pertaining to the phenotypic characteristics of cancer cells such as division, invasion or metastatic potential. Interestingly, several authors have pointed out that a correlation between coronaviruses such as the SARS-CoV-1 and -2 or MERS viruses and dysregulations of signaling pathways activated by TKRs can be established. This information may help to accelerate the repurposing of clinically developed anti-TKR cancer drugs in COVID-19 management. Because the need for treatment is critical, drug repurposing may be an advantageous choice in the search for new and efficient therapeutic compounds. This approach would be advantageous from a financial point of view as well, given that the resources used for research and development would no longer be required and can be potentially redirected towards other key projects. This review aims to provide an overview of how SARS-CoV-2 interacts with different TKRs and their respective downstream signaling pathway and how several therapeutic agents targeted against these receptors can interfere with the viral infection. Additionally, this review aims to identify if SARS-CoV-2 can be repurposed to be a potential viral vector against different cancer types.

## 1. Introduction

Coronaviruses are RNA viruses that affect mammals, having an affinity for the respiratory apparatus in humans. Strains of coronavirus, namely severe acute respiratory syndrome coronavirus (SARS-CoV) and Middle East respiratory syndrome coronavirus (MERS-CoV), have previously caused a large number of cases before completely disappearing. SARS-CoV-2’s origin is currently still unknown, but bats are a very likely source, as SARS-CoV and MERS-CoV, similar coronaviruses, have been associated with bats [1,2]. SARS-CoV-2 and bat-CoV RaTG13 share a 96.2% genome sequence identity, demonstrating a common ancestry between the two viruses [3]. The incidence of COVID-19, the infectious disease caused by SARS-CoV-2, is constantly increasing, with almost 62 million confirmed cases and almost 1.5 million deaths worldwide. SARS-CoV-2’s human-to-human transmission is mainly sustained through direct contact or through coughing and sneezing droplets received from an infected individual [4].

SARS-CoV-2 is the newest strain of beta coronaviruses, known to have an incubation period of 5.2 days [5]. However, cases with longer incubation periods, up to 24 days, have been reported [6]. This long incubation period, through which the patients present no symptoms but are contagious, is considered one of the main reasons why SARS-CoV-2 has spread so fast around the world [5]. After this asymptomatic period, the symptoms that usually appear are the following: fever, fatigue, cough, headache, difficulty in breathing, hemoptysis, sputum production, sore throat and diarrhea [7,8].

The pathogenesis of the virus is mainly represented by the attachment of the spike (S)-glycoprotein located on the surface of the coronavirus to the angiotensin conversion enzyme 2 (ACE2) receptor from the human cells [9]. The S-glycoprotein is composed of two subunits, S1 and S2. S1’s main purpose is determining the virus–host range and cellular tropism with the key function domain, the receptor-binding domain (RBD), while S2 mediates virus–cell membrane fusion through two tandem domains, heptane repeats (HR) 1 and 2 [10].

Furthermore, research has been conducted regarding the ability of the SARS-CoV-2 S1 RBD to bind heparin. Heparins are drugs used for their anticoagulant/thrombotic properties and are known for being safe, stable and highly effective. They also present antiviral activity, which was never fully explored in a clinical setting. Interestingly, coronaviruses are also targeted by heparin because of SARS-CoV’s envelope proteins containing positively charged amino acids that are prone to interact with the negatively charged sulfate groups of heparin sulfate proteoglycans [11].

The innate immune system is activated, and pattern recognition receptors (PRRs) are used to recognize the pathogen-associated molecular patterns (PAMP). PRRs consist predominantly of toll-like receptor (TLR), RIG-I-like receptor (RLR) (also previously demonstrated in MERS-CoV [12]), NOD-like receptor (NLR), C-type lectin-like receptors (C_Lmin_) [13], cytosolic receptor melanoma differentiation-associated gene 5 (MDA5) and nucleotidyl transferase cyclic GMP-AMP synthase (cGAS) [14].

The aforementioned complex factors catalyze the activation of the transcription factor nuclear factor-κB (NF-κB) and interferon regulatory factor 3 (IRF3), leading to the production of type I interferons (IFN-α/β) and a series of proinflammatory cytokines [15,16].

Oncolytic virotherapy is a novel therapy consisting of the use of replicating viruses, through the genetic modification that they produce in cells, as a means of treating cancer. The viruses’ tropism is restricted in order to infect only certain cell types. Furthermore, exogenous genes can be added in order to make the virus more aggressive, hence inducing the host’s immune response against the specifically targeted cancer cells [17,18].

## 2. Growth Factors, Tyrosine Kinase Receptors and SARS-CoV-2: A Complex Equation

**Figure 1:**
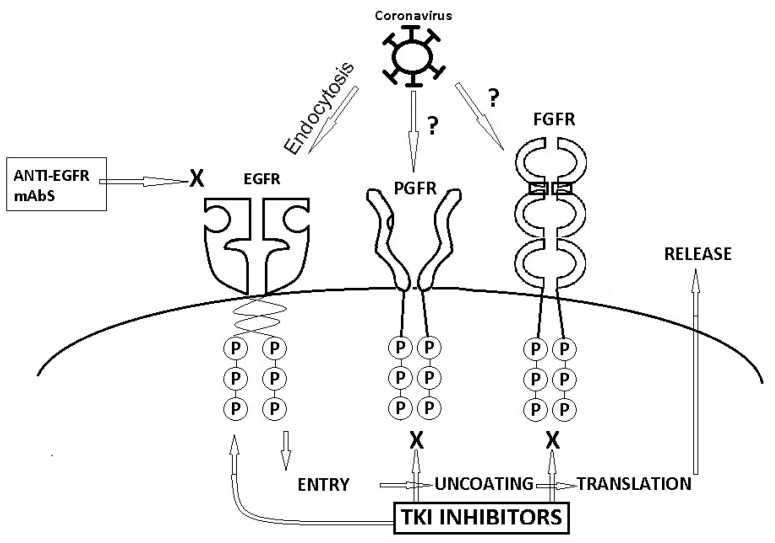
The potential role of several GFRs in coronavirus infection.

Growth factor receptors (GFRs) possess the important role of binding extracellular polypeptide growth factors, which determines a cascade of signaling events with the final purpose of regulating cell growth [19]. GFRs are also relevant for the entry of multiple viruses, including coronaviruses, which makes them a central topic of discussion regarding the SARS-CoV-2 pandemic. Drugs inhibiting GFRs that are used for antitumoral purposes are presented in Figure 1 [20,21,22].

### 2.1. The Epithelial Growth Factor Receptor

The epidermal growth factor receptor (EGFR) is a member of the ErbB family of TKRs with important functions in epithelial cell physiology [23]. It is well known for presenting overexpressions and mutations in a multitude of human cancers, hence becoming the target for multiple cancer therapies [24]. EGFR tyrosine kinase inhibitors (TKIs) have been well documented in numerous clinical studies and are used in the treatment of several types of cancer, most notably non-small-cell lung cancer (NSCLC), for almost two decades now [25].

The EGFR can play a role in the internalization of coronaviruses through binding to the S protein. Transmissible gastroenteritis virus (TGEV) is an alpha-coronavirus that infects the epithelial cells of the intestine, causing severe, potentially lethal, diarrhea in piglets. In a study, the mechanism of infection of TGEV was analyzed, concentrating on the binding with the EGFR. The internalization of the virus was achieved through clathrin- and caveolin-mediated endocytosis. Afterwards, the virus was bound to the EGFR, promoting successive clathrin-mediated endocytosis [26].

After the TGEV spike protein binds with EGFR, the phosphoinositide 3 kinase (PI3K) pathway is activated, inducing the phosphorylation of cofilin and the polymerization of F-actin via Rac1/Cdc42 GTPases. EGFR activates the MAPK pathway, correlated with F-actin reorganization, thus proving again the important involvement of the EGFR in the coronavirus endocytosis [27]. TGEV infection can be also treated with A9, a TKI of the tyrphostin class. In a preclinical in vitro study, the A9 inhibitory activity of the TGEV was mediated by the p38 MAPK signaling pathway. A study by Dong et al. proved the potential of targeting p38 as a means of treating coronaviruses [28].

Researchers explored the possibility of how SARS-CoV infection can influence EGFR signaling and consequently amplify the effect of the receptor’s activation. The authors tested the potential of EGFR to provoke fibrosis and how much it varies depending on the presence of viral infection. To their surprise, the overregulation of EGFR signaling followed by SARS-CoV infection determined higher levels of inflammation in the lungs than it would be normally expected alongside interstitial edema [29].

EGFR TKIs are known to have the side effect of promoting interstitial lung disease in the patients receiving these drugs [30]. An important similarity between this interstitial lung disease and the characteristics of COVID-19 has been observed, from the clinical symptoms (fever, cough, fatigue, sputum production, shortness of breath, myalgia, etc.) to radiological findings (ground-glass opacities) [31].

Gefitinib, a TKI used for the first-line treatment of EGFR-mutated NSCLC for almost two decades, is known to aggravate pulmonary fibrosis inflicted by bleomycin [32,33,34]. Amphiregulin, a ligand of the EGFR, encoded by the AREG gene, is upregulated in many cancers, determining cell growth, proliferation and migration through major intracellular signaling pathways triggered by receptor binding. In murine models, silencing amphiregulin by siRNA or using EGFR-specific TKIs attenuated the fibrogenic effects of TGF-beta1, TGF-beta1 being known for its fibrosis-inducing characteristics [35]. In another study, it was shown that TGF-alpha-mediated fibrosis can be prevented by treating mice with gefitinib and erlotinib [36].

The available data are contradictory given that anti-EGFR TKIs can cause pulmonary fibrosis in humans while preventing pulmonary fibrosis in mice, so there can be several ways of explaining the difference. One explanation is that EGFR signaling can determine different downstream results, depending on the species it encounters. Another way of explaining this discrepancy is that the EGFR downstream signaling kinetics could be dysregulated and not necessarily dependent on the strength of the signal itself [37].

### 2.2. The Fibroblast Growth Factor Receptor

Fibroblast growth factor receptors (FGFRs) are TKRs that possess an important role in cell proliferation, migration and differentiation. The dysregulation of their expression can lead to the emergence of different tumors [38]. The FGFR family has four members: FGFR1-4, encoded by different genes but presenting high homology [39]. FGFR TKIs are becoming an important tool in the inhibition of cancer growth, with multiple clinical trials assessing the effectiveness of anti-FGFR TKIs [40].

FGFR can also be relevant in viral infections. FGF, bound to heparan sulfate molecules, interacts with FGFR, creating a trimolecular FGF–HS–FGFR complex, setting off subsequent FGFR activation [41]. FGFR1 was proven to be an important, indispensable cofactor in infection with adeno-associated virus 2. Viral invasion was thought to be regulated by heparan sulfate proteoglycans alone, but it was later understood that both HSPG and FGFR1 were implicated in the endocytosis of the virus [42]. FGFR was also relevant in influenza virus infection, being a cofactor necessary for the early stages of the infection [43].

In a study by Hardie et al., several human kinases were screened in order to identify those that could be linked to dengue fever replication. Of those explored, the study focused on the role of FGFR4, a member of the FGFR family. The study showed that dengue fever infection determines an impairment of FGFR phosphorylation. More interestingly, the inhibition of FGFRs via siRNA provided a decrease in the RNA replication of dengue virus, while simultaneously increasing its viral particle production, suggesting that the FGFR might play a regulatory role in the lifecycle of the virus, switching between the early and late stages [44]. In another study, FGF2 was blocked in a Zika-virus-infected human astrocyte cell culture to see how it affects viral replication. The study showed that treatment with the monoclonal antibody BGJ398 determined a decrease in viral replication and cell-to-cell transmission, mainly through the inhibition of the MAPK pathway, which is strongly linked to normal FGF/FGFR activity [45]. In another study, the association of Epstein–Barr virus (EBV) with nonkeratinizing nasopharyngeal cancer (NPC) was explained through the perspective of FGFR1 signaling in the LMP1 pathway. FGFR1 inhibition managed to suppress cell multiplication, migration and invasion in the NPC. Aerobic glycolysis and the epithelial cell transformation demonstrated the association between FGFR/FGF2 signaling present in the EBV activity and the NPC [46].

Another study that analyzed MERS-CoV-induced apoptosis in kidney and lung tissues discovered a correlation between FGFR2 inhibition and the degree of cell death induced by viral infection. By using a specific anti-FGFR TKI, tyrphostin AG1296, the authors observed a reduction in apoptosis by over 40%. However, an anti-EGFR tyrphostin, AG490, had no influence over MERS-CoV-induced apoptosis [47].

### 2.3. The Platelet-Derived Growth Factor Receptor

Platelet-derived growth factor receptors (PDGFRs) are TKRs with important functions in the development of connective tissue. The two types of receptors are PDGFRα and PDGFRβ. PDGF-PDGFR signaling is important in development, but in the adult age, its function remains relevant only in tissue repair and lesion healing [48]. The most mainstream PDGFR inhibitors are TKIs, with the vast majority of them being nonspecific, targeting additional structures involved in cancer development such as KIT and FLT3 [49].

It was shown that influenza virus entered the cell through the PDGFRβ/GM3 signaling pathway, and endocytosis was successfully inhibited with the TKIKi8751, which specifically targets PDGFRβ phosphorylation. [50]. Furthermore, it was also discovered that PDGFRα plays an important role in the entry of cytomegalovirus into fibroblasts. Through a genome-wide CRISPR screen, PDGFR was shown to have the most significant role in trimer-only human cytomegalovirus (HCMV) infection [51]. Moreover, in a similar study, the silencing of PDGRα reduced the spread of gH/gL/gO-positive HCMV, demonstrating PDGRα’s essential role in cell endocytosis [52]. Contrastingly, in another study, it was shown that PDGFRα was not involved in the HMCV entry of the trimer, its silencing producing no effect on the virus endocytosis [53].

## 3. The Link between Antiviral and Anticancer Drugs

Anticancer drugs have consistently shown potential in the treatment of antiviral infections. During the SARS-CoV-2 pandemic, an important task for researchers has been to find a correlation between the antiviral and antineoplastic function of drugs in order to implement them most effectively in the treatment protocols of COVID-19 patients [54]. Even more so, oncological treatment during the SARS-CoV-2 pandemic is more difficult than ever, as cytotoxic therapies have side effects, such as leukopenia, which makes the organism highly susceptible to infections [55].

Ibrutinib, a powerful inhibitor of the Bruton tyrosine kinase (BTK), is a drug that has a possible anti-inflammatory effect best observed in the respiratory apparatus. Its ability to reduce lung damage, cytokine levels in the lung tissue and mortality have been documented in animal experiments using the H1N1 influenza virus strain. The animals that received ibrutinib survived and made a complete recovery [56].

The effect of ibrutinib was also tested in SARS-CoV-2 subjects. A total of 300 patients suffering from Waldenström’s macroglobulinemia (WM) were included in a study in which they received BTK inhibitors. Six of these patients were diagnosed with a SARS-CoV-2 infection and received different doses of ibrutinib (five of the patients received 420 mg/day, while only one patient received 140 mg/day). Patients receiving the higher dose presented better evolution with easier symptoms and with hospitalization not being necessary. On the contrary, patients receiving the lower dose showed symptoms with increasing severity, which caused the necessity of hospitalization [57].

Acalabrutinib, another BTK inhibitor, was also successful in the treatment of several patients suffering from severe cases of COVID-19. The patients, 11 of whom received supplemental oxygen and 8 of whom were on mechanical ventilation, were administered acalabrutinib, with improved oxygenation being observed for the majority of them. This proved that BTK inhibitors are relevant for targeting excessive host inflammation in COVID-19 patients [58].

Selinexor, a selective inhibitor of nuclear export (SINE), is a drug approved for treating relapsed/refractory multiple myeloma [59]. SINEs are known to have the ability to reduce viral proliferation and thus were used in a clinical trial for patients suffering from COVID-19. The drug managed to inhibit important host–protein interactions for SARS-CoV-2 [60].

### The Role of Tyrosine Kinase Inhibitors in the Treatment of Coronavirus Infections

TKIs are considered a potential treatment for COVID-19, as they are known to target specific host functions that are required by multiple viruses, including SARS-CoV-2 [61].

MAPK/ERK and PI3K/AKT/mTOR signaling responses have been shown to be relevant in MERS-CoV infection through bioinformatics analysis in vivo. Therefore, by suppressing these pathways, the replication was substantially inhibited in vitro [62].

For SARS-CoV, the potential for use of imatinib, an ABL 2 inhibitor approved for clinical practice 20 years ago, was attested due to the inhibition of the replication of SARS-CoV and MERS-CoV prior to RNA production. Thus, a correlation was found between Abl2 and the productive replication of SARS-CoV and MERS-CoV [63]. Imatinib can be useful for treating pneumonia associated with SARS-CoV-2 infection, as it has been proven to be efficient in treating pulmonary diseases [64]. It improved patients with pulmonary and systemic vascular leak [65], severe refractory asthma [66] and pulmonary artery hypertension [67]. On the contrary, it did not improve patients with idiopathic pulmonary fibrosis [68].

For the more recent SARS-CoV-2, imatinib’s viral inhibiting properties have been tested in vitro and results showed potential for inhibition, acting especially on the spike protein and blocking the viral entry at the endosomal level [69]. Prostaglandin E2 stimulation and the deceleration of the increase in TNF-α, IL1-β and IL-6 were observed in the case of administering imatinib, thus reducing inflammation. Imatinib has been proven to interfere in the NF-κB signaling pathway, suppressing it [70]. This pathway is activated in SARS-CoV-2-infected patients and is believed to facilitate the activity of the virus [71]. There have been attempts for treatment with imatinib. In a study, imatinib was added to the treatment protocol at the same time with the interruption of ceftriaxone. Astonishingly, the fever disappeared, the supplementation with oxygen was ceased and pulmonary stability was radiologically confirmed [72].

The possible link between JAK inhibitors (JAKi) and SARS-CoV-2 has also been taken into account. JAKi are drugs that usually have a tendency to interfere with the immune system, increasing the infectious risk in patients. There have been three anecdotal cases of patients that tested positive for SARS-CoV-2 who are taking JAKi for alopecia areata. None of them had significant events but were nonetheless taken off JAKi. Thus, an important aspect during the pandemic is that doctors are careful what drugs they are prescribing to their patients, especially if those drugs have a potential influence on the evolution of COVID-19 [73].

## 4. SARS-CoV-2 and Viral Tumorigenicity: A Tale of a Two-Edged Sword

### 4.1. SARS-CoV-2-Induced Carcinogenesis via Tyrosine Kinase Receptors

The carcinogenic potential of viruses is a well-known and documented fact. Of the 219 viral species known to humans, almost 150 types of viruses have carcinogenic potential. Some, such as HPV, are exceptionally carcinogenic, being responsible for almost 95% of cervical cancer cases, while others, such as human herpesvirus 8, are linked to rarer types of cancer such as Kaposi’s sarcoma [74]. While the number of coronaviruses is quite vast, very little information is available on their carcinogenic potential as of yet. A preclinical model analysis suggested that SARS-CoV-2 presents a very high affinity for EGFR, VEGFR and c-MET receptors present on glial cells, which are strongly related to gliomagenesis [75]. However, can SARS-CoV-2 penetrate the blood–brain barrier (BBB)? Given the large number of neurological symptoms described by COVID-19 patients, it was strongly suggested that the virus is capable of easily penetrating the BBB. In a study by Rhea et al., it was demonstrated that a radioiodinated S protein can freely traverse the BBB in murine models [76]. Another study has shown that SARS-CoV-2 is capable of infecting the choroid plexus, strongly disrupting the BBB [77].

Another relation between SARS-CoV-2 and TKR activity in cancer might be established between the large number of proinflammatory cytokines and chemokines, which are largely responsible for acute respiratory distress syndrome and the tumor microenvironment, which has a strong impact on carcinogenesis; more explicitly, a link between the IL-6/JAK/STAT3 pathway and the plethora of proinflammatory molecules found in patients suffering from COVID-19. For example, Zhang et al. observed that the mortality of bladder cancer patients suffering from COVID-19 was 10 times higher than that of other patients suffering from the virus [78]. This was theorized to be related to the activation of the IL-6/JAK/STAT3 pathway in the tumor microenvironment of bladder cancer patients, which further exacerbates the inflammation caused by COVID-19 [79]. Another example of the link between the tumor microenvironment and COVID-19 is found in ovarian cancer. Ovarian cancer is known to present increased levels of IL-2, IL-6, IL-12 and Il-13, while high levels of IL-6 are frequently encountered in COVID-19. The interaction between IL-6 and sIL-6R has been shown to promote ovarian cancer progression through the ERK, a TK that triggers increased cell survival, migration and invasion [80]. These mechanisms indicate that high levels of proinflammatory cytokines during the COVID-19 infection could act as a trigger for cancer development and progression, mediated by signals initiated through TKRs or downstream signaling pathways shared with TKRs.

### 4.2. Oncolytic Virotherapy Potential of the Coronavirus

Oncolytic virotherapy is becoming an attractive option for the treatment of patients with different forms of cancer. Several clinical studies have researched the use of viral therapy, providing promising results [81,82,83]. Coronaviruses have been researched in this particular subject as potentially capable of exerting an antitumoral effect. The virus has to be modified with an additional protein, or antibody, in order to direct them against the EGFR, thus creating a tumor-targeting virus. [84].

The infectious properties of mouse hepatitis coronavirus (MHCV) are obtained through the binding of its S protein to the murine carcinoembryonic antigen-related cell adhesion molecule 1 (CEACAM1a). Virus–cell membrane fusion is achieved through the induction of conformational changes after the binding of the N-terminal part of the cellular receptor (soR) and the S protein. In order to direct it against the EGFR, a single-chain monoclonal antibody 425 was bound to the soR, creating a bispecific adapter protein (soR-425). The soR-425 proved successful in targeting the EGFR in vitro, but the S protein fusion process was necessary for the virus entry. This research first demonstrated the potential of coronaviruses for tumor-targeting purposes [85].

A few years later, a similar experiment was performed and was again successful in vitro and subsequently continued with an in vivo study. A mouse previously exposed to a lethal intracranial tumor was treated with an MHCV soR-EGF (adaptor protein soR-EGF injected into the MHCV’s genome) injection. This significantly prolonged its survival, stopping the recurrence of the tumor load [86].

## 5. Discussion

The EGFR and other TKRs seem to have a strong correlation with SARS-CoV-2, providing diverse insights into the treatment of COVID-19. The direct binding to the EGFR of TGEV shows that coronaviruses have an affinity for the EGFR, so a potential application for the future is blocking the endocytosis at this level by downregulating the signaling pathway that promotes it. TKIs, more specifically A9 (a tyrphostin-class TKI), produced a satisfactory response in vitro, partially inhibiting the endocytosis of the virus through the EGFR.

A common aspect between SARS-CoV-2 and the EGFR TKIs is that they promote interstitial lung disease, having a high similarity of symptoms and radiological showings. Furthermore, EGFR overexpression facilitates pulmonary fibrosis for a SARS-CoV-infected patient. Although many studies have offered the perspective that EGFR has antifibrotic properties, there has also been research demonstrating the opposite. The difference may come from the different species involved in the testing or from the fact that the signal’s intensity/time is not relevant for the EGFR’s activity in fibrosis.

Imatinib, a representative of the TKI class, has proven efficient in the inhibition of replication in SARS- and MERS-CoV, proving the implication of Abl2 in the replication. Imatinib was recently tested for SARS-CoV-2 and it successfully inhibited the endocytosis of the virus and also suppressed the NF-κB signaling pathway, which enhanced viral activity levels. The importance of JAKi for COVID-19 has been questioned on a theoretical level, JAKi being known to negatively affect the immune response. The purpose of this correlation is that during the pandemic, the prescription of drugs that may have an effect on the immune response of patients should be closely regulated in order to minimize the rampant advancement of the pandemic.

The EGFR and SARS-CoV-2 correlation is also relevant in the oncology field. The potential for oncolytic virotherapy is an important one. Coronaviruses can exert an antitumoral effect when attached to the cancer cell. The targeting of the cell is achieved through the modification of the virus. An additional protein or antibody is bound to the virus, making it prone to connect to the EGFR of the cancer cell.

Experiments have been conducted for coronaviruses (e.g., mouse hepatitis coronavirus) and proved successful in vitro and in vivo. The use of bispecific adapter proteins attached to the virus redirected its course towards the EGFR, and endocytosis occurred through S protein fusion.

SARS-CoV-2 can certainly be relevant in the oncolytic virotherapy approach, as per its similarity with the MHCV coronavirus, with tests and research required in order for SARS-CoV-2 to prove itself as an important candidate for effective tumor targeting and cancer treatment.

No treatment has proven successful in treating SARS-CoV-2 as of yet. With the race to implement an international immunization scheme through vaccination being strongly underway, it might prove wise to try to replicate different treatment strategies that proved effective for other types of viral agents. Additionally, with oncolytic viral therapy being a popular option in the last decade, SARS-CoV-2 might prove useful as a therapeutic agent for the treatment of different cancer forms.

## Abbreviations

TKR	Tyrosine kinase receptor
SARS-CoV	Severe acute respiratory syndrome coronavirus
MERS-CoV	Middle East respiratory syndrome coronavirus
ACE2	Angiotensin conversion enzyme 2
RBD	Receptor-binding domain
HR	Heptane repeats
PRRs	Pattern recognition receptors
PAMP	Pathogen-associated molecular patterns
TLR	Toll-like receptor
RLR-RIG	I-like receptor
NLR-NOD	Like receptor
CLmin-C	Type lectin-like receptors
MDA5	Cytosolic receptor melanoma differentiation-associated gene 5
cGAS	Nucleotidyltransferase cyclic GMP-AMP synthase
NF-κB	Transcription factor nuclear factor-κB
IRF3	Interferon regulatory factor 3
IFN	Interferon
GFR	Growth factor receptors
EGFR	Epithelial growth factor receptor
NSCLC	Non-small-cell lung cancer
HMCV	Human cytomegalovirus
TKI	Tyrosine kinase inhibitors
PI3K	Phosphoinositide 3 kinase
TGEV	Transmissible gastroenteritis virus
FGFR	Fibroblast growth factor receptor
EBV	Epstein–Barr virus
NPC	Non-keratinizing nasopharyngeal cancer
PDGFR	Platelet-derived growth factor receptors
BTK	Bruton tyrosine kinase
SINE	Selective inhibitor of nuclear export
MHCV	Mouse hepatitis coronavirus
CEACAM1a	Carcinoembryonic antigen-related cell adhesion molecule 1
JAKi	JAK inhibitors
BBB	Blood–brain barrier
